# Laboratory Capacity Building in Asia for Infectious Disease Research: Experiences from the South East Asia Infectious Disease Clinical Research Network (SEAICRN)

**DOI:** 10.1371/journal.pmed.1000231

**Published:** 2010-04-06

**Authors:** Heiman F. L. Wertheim, Pilaipan Puthavathana, Ngoc My Nghiem, H. Rogier van Doorn, Trung Vu Nguyen, Hung Viet Pham, Decy Subekti, Syahrial Harun, Suhud Malik, Janet Robinson, Motiur Rahman, Walter Taylor, Niklas Lindegardh, Steve Wignall, Jeremy J. Farrar, Menno D. de Jong

**Affiliations:** 1 South East Asia Infectious Diseases Clinical Research Network; 2 Oxford University Clinical Research Unit, National Hospital of Tropical Diseases, Hanoi, Vietnam; 3 Centre for Tropical Medicine, Nuffield Department of Clinical Medicine, Oxford University, Oxford, United Kingdom; 4 Siriraj Hospital, Mahidol University, Department of Microbiology, Bangkok, Thailand; 5 Oxford University Clinical Research Unit, Hospital of Tropical Diseases, Ho Chi Minh City, Vietnam; 6 National Hospital of Tropical Diseases, Hanoi, Vietnam; 7 National Hospital of Pediatrics, Hanoi, Vietnam; 8 South East Asia Infectious Disease Clinical Research Network (SEAICRN) Coordinating Center, Eijkman Institute, Jakarta, Indonesia; 9 National Institute of Health Research and Development, Jakarta, Indonesia; 10 Family Health International, Bangkok, Thailand; 11 Mahidol-Oxford Research Unit, Mahidol University, Bangkok, Thailand; 12 Academic Medical Center, Amsterdam, The Netherlands

## Abstract

Heiman Wertheim and colleagues discuss a network that aims to improve infectious disease management through integrated, collaborative clinical research in South East Asia.

**Summary Points**

- Enhancing laboratory capacity is essential for generating reliable and accurate data from clinical research, especially in resource-constrained settings.
- Local well-trained laboratory experts and scientists are important to research, and must participate actively in scientific activities and continuing education programs.
- Improving laboratory capacity is more than supplying new equipment and reagents; it also includes a long-term commitment to staff training, quality control, and biosafety.
- Improved laboratory capacity optimizes responses to an epidemic or an outbreak of a novel virulent pathogens, and can support international agendas to reduce the impact of pandemic influenza viruses.

## Introduction

The South East Asia Infectious Disease Clinical Research Network (SEAICRN) is a collaborative partnership of hospitals and institutions in Thailand, Vietnam, Indonesia, and Singapore. The network strives to advance scientific knowledge and clinical and laboratory management of infectious disease in general, and influenza in particular, through integrated, collaborative clinical research in the South East Asia region (see http://www.seaicrn.org/) [1]. The establishment of this network helps fulfill the declaration of the World Health Assembly in 2005 that urged its member states to “strengthen national laboratory capacity for human and zoonotic influenza,” and “to support an international research agenda to reduce the spread and impact of pandemic influenza viruses” [2],[3]. In addition it also supports the recently revised International Health Regulations (IHR 2005), which came into effect in 2007, by increasing surveillance and response capacity in strategic hospitals, national institutes, and referral laboratories and by promoting international collaboration and sharing of data [3].

SEAICRN's first intervention study was a multicenter randomized clinical trial in four Asian countries that compared the efficacy of high dose (150 mg bid) versus standard dose (75 mg bid) oseltamivir for severe influenza (Table 1). Subsequently the SEAICRN initiative developed a series of research protocols related to influenza and other infectious diseases being implemented in participating institutions. To make these studies possible, and to comply with international laboratory and clinical trial standards, the SEAICRN recently chose to enhance the capacity and quality of both the research and clinical laboratories at all participating hospitals and institutions in the region.

**Table 1:** List of approved protocols that will be or are being executed by SEAICRN.

Study Number	Clinical Trial Registry Number	Title	Status
SEA001	NCT00298233	High-dose versus standard-dose oseltamivir for the treatment of severe influenza and avian influenza. A phase ii double-blind, randomized clinical trial	Study finished. Awaiting analysis
SEA002	NCT00439530	Pharmacology Study of oseltamivir and probenecid in healthy Asian subjects	Published
SEA003	NCT00921726	Phase 1, open-label study to evaluate potential pharmacokinetic interactions between orally-administered oseltamivir and intravenous zanamivir in healthy Thai adult subjects	Study finished. Awaiting analysis
SEA004	NCT00980109	Long term influenza prophylaxis with inhaled zanamivir or oral oseltamivir	Ongoing
SEA005	Pending	An open label study to evaluate the safety, tolerability and efficacy of intravenous zanamivir administered twice daily for 5 days in hospitalized subjects with confirmed H5N1 influenza infection.	Will start 2010
SEA006	—	Clinical database of patients with H5N1 infection	Ongoing
SEA007	—	Host genetic susceptibility to human influenza A/H5N1	Ongoing
SEA008	—	Cardiorespiratory function and radiological changes in lung structure in Indonesian patients who survive highly pathogenic influenza A/H5N1—A 3 year prospective study	Ongoing
SEA013	Pending	Open-label study to evaluate potential pharmacokinetic interactions between orally administered oseltamivir and intravenous peramivir in healthy Thai adult subjects	Tentatively scheduled after January 2010
SEA019	—	Non-Influenza etiologies of acute respiratory illness in Southeast Asia	Ongoing
SEA022	Pending	Oseltamivir treatment in severe, virologically confirmed influenza A or B in hospitalized children less than one year of age in Hanoi, Ho Chi Minh City and Bangkok	Expected to start in 2010
SEA023	—	The viral etiology of hospitalized children less than one year of age with an acute, community acquired, lower respiratory tract infection in Hanoi and Bangkok	Expected to start in 2010
SEA024	Pending	Efficacy of primaquine against relapse by *Plasmodium vivax* in Indonesia	Expected to start in 2010
SEA025	—	A survey of acute febrile illnesses in adults and children at the Cipto Mangunkusumo hospital, Jakarta, Indonesia	Expected to start in 2010
SEA026	—	Characterization of the human carboxylesterase 1 (CES 1) mutation(s) which may be responsible for markedly reduced conversation of oseltamivir phosphate to oseltamivir carboxylate, and determination of frequency in SE Asian population	Ongoing
SEA027	—	Acute post-infectious measles encephalitis	Finished. Awaiting analysis
SEA028	—	Hand, foot, and mouth disease	Protocol in development
SEA029	Pending	A randomized, open-label, comparative trial of the efficacy of itraconazole versus Amphotericin B in the treatment of penicilliosis in HIV-infected persons	Will start beginning of 2010
SEA030	Pending	An open label study to evaluate the safety, tolerability and efficacy of intravenous zanamivir administered twice daily for 5 days in hospitalized subjects with confirmed seasonal influenza infection	Pending
SEA032	NCT00985582	Oseltamivir treatment in adults and children with mild or moderate influenza H1N12009 infection—a clinical, virological and pharmacokinetic study	Ongoing

Study SEA002 is finished and already published [13].

The key objective of the laboratory strengthening program was to enhance laboratory facilities; ensure availability of necessary equipment; build human resource capacity by teaching, training, and mentoring; and ensure quality laboratory management and testing to comply with good clinical laboratory practice (GCLP) and other international standards such as the ISO 15189 [4],[5]. Here we present the experiences of the SEAICRN in setting up and conducting the laboratory capacity building program and discuss how this benefitted the institutions, the research studies, and the region. The US National Institutes of Health's (NIH) National Institute for Allergy and Infectious Disease (NIAID) and Wellcome Trust funding support for this program has been described previously [1].

## Creating Laboratory Capacity

**Figure 1:**
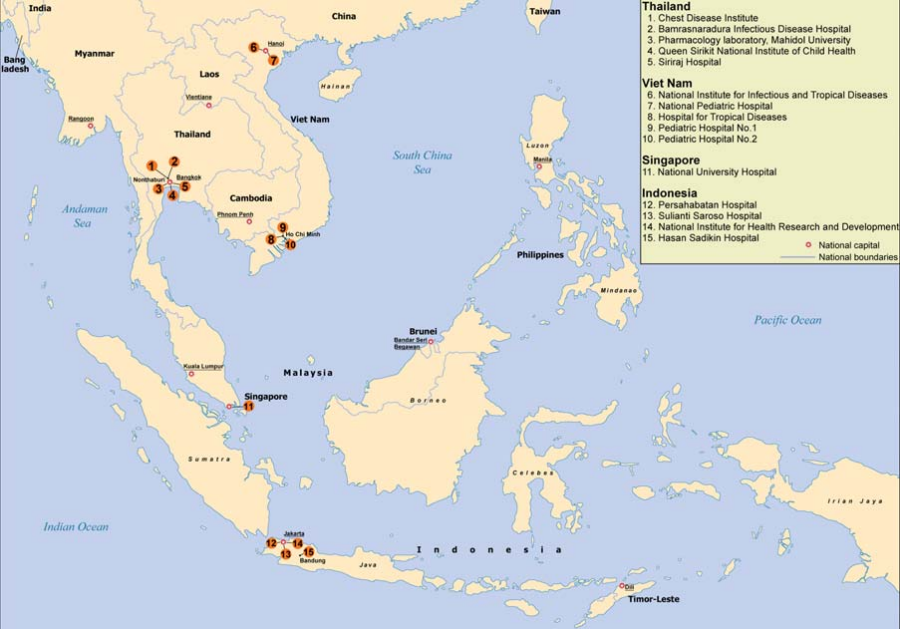
South East Asia Infectious Disease Clinical Research Network sites and laboratories.

Given that SEAICRN laboratories had different levels of organization and expertise, it was considered important to create reference laboratories for different aspects of the research activities in each country (Figure 1). Due to the initial focus on influenza therapeutics, a SEAICRN clinical pharmacology laboratory was established at the Department of Tropical Medicine, Mahidol University, Bangkok, Thailand.

To enroll patients into the first clinical trial, participating sites needed to be able to screen patients by influenza reverse transcriptase polymerase chain reaction (RT-PCR) reliably, rapidly, and accurately. To be able to screen patients by RT-PCR, several laboratories were renovated to house, for the first time, a molecular diagnostic laboratory (MDL), with all the required infrastructure, equipment, reagents, and consumables. Physical separation of spaces for reagent preparation, nucleic acid extraction, and amplification was ensured in all laboratories to prevent PCR contamination. Also, a new biosafety level 3 laboratory facility was established at the Hospital for Tropical Diseases, Ho Chi Minh City, Viet Nam for isolation of H5N1 viruses and emerging (pandemic) influenza viruses. Additionally, in Viet Nam, (pyro)sequencing capacity was established to characterize viruses and detect mutations in the influenza virus genome that confer antiviral drug resistance.

Site staff from Bangkok, Jakarta, Bandung, Hanoi, and Ho Chi Minh City were trained in influenza real-time RT-PCR diagnostics and molecular diagnostics in general, including principles of unidirectional workflow and prevention of carry-over contamination. These trainings were performed on an ongoing basis at the reference laboratory in Ho Chi Minh City, followed by on-site training. To ensure quality, each MDL laboratory was enrolled in at least two different external quality assurance programs for molecular influenza diagnostics. Furthermore, proficiency testing for diagnosing influenza by RT-PCR was performed before sites were allowed to start screening patients for the studies.

Each hospital is encouraged to use the MDL for purposes other than SEAICRN-related research activities, such as HIV, hepatitis, dengue, meningitis, and encephalitis testing by molecular techniques. The philosophy is that it is important and highly beneficial to increase expertise in detecting infectious agents by molecular diagnostics and so stimulate the implementation of other relevant molecular diagnostic tests. For example, after implementation of a *Streptococcus suis*–specific molecular test in the MDL of the National Hospital of Tropical Diseases, Vietnam, it was found that *S. suis* is a common cause of bacterial meningitis in adults in Hanoi [6].

In preparation for the start of the clinical trials, dedicated laboratory trial staff members were appointed to become staff of the national institutions. They received training on processing, labeling, testing, storing, shipping, and documenting data from trial specimens according to protocol requirements. A centralized specimen labeling and database system was set up to track and trace all stored specimens for the SEAICRN studies. Currently the SEAICRN uses Freezerworks software (Dataworks Development; http://www.freezerworks.com/) to track study specimens and aliquots. Sites received deep freezers for adequate specimen storage and power back-up systems were installed where needed to ensure an uninterrupted power supply. Table 2 summarizes the required laboratory capacity and what was done to achieve this.

**Table 2:** Activities by SEAICRN to achieve required laboratory capacity for their research projects.

Context	Capacity/Expertise Required	Activity
***Infrastructure***	Uninterrupted power supply	UPS systems installed
	Sufficient freezer capacity for study specimen storage	Provided freezers
	MDL	Create MDL where needed and train staff.
***Quality and safety***	External quality assurance (EQA) of laboratory tests	Provided EQA (Thai program)
	Equipment maintenance	Ensured maintenance
	Temperature logs	Set up temperature logging system
	Laboratory safety	Provide safety cabinets and biosafety training
	Laboratory test normal ranges	Provided guidance in establishing normal ranges
	Laboratory result documentation	Improved where needed
	Laboratory accreditation	Laboratory quality enhancement program implemented
	Specimen/aliquot tracking and tracing	Implemented a central labeling system and database
	Shipment of infectious substances	Training in shipping biological substances
***Specific laboratory testing***	Influenza molecular diagnostic test	Set up CDC assay for influenza real-time RT-PCR
	Neuraminidase inhibitor resistance testing	Set up neuraminidase inhibition assay and molecular based test (sequencing)
	Oseltamivir plasma concentrations	Set up pharmacokinetic laboratory
	Laboratory tests for adverse events	In case unavailable, set up referral system.
	Blood culture	Provided blood culture system
***Other***	Listen for local needs and challenges and provide technical assistance when able to do so.	Provided technical assistance and support as part of the program

The laboratory capacity program of the SEAICRN enabled us to rapidly respond to changes in influenza epidemiology, such as the spread of naturally occurring oseltamivir resistance in seasonal H1N1 viruses and the recent emergence of the novel influenza A (H1N1) strain (pH1N1) in April 2009. In response to the increasing rates of oseltamivir resistance in seasonal H1N1 viruses, before the emergence of pH1N1, the laboratory committee decided to test Asian isolates and respiratory specimens from influenza patients in our studies [7],[8]. This step generated valuable resistance data from the region, which were instantly shared with the national and international medical and scientific community. In April 2009, when confronted with a pandemic threat of this novel influenza virus, we prepared collaborating laboratories by providing them with sequence information of this strain and primers and probes to enable them to detect it by RT-PCR. Furthermore, we were able to describe the first worldwide transmission of oseltamivir resistant pH1N1 in a community cluster [9].

## Laboratory Quality Enhancement Program

In partnership with Family Health International (FHI), the SEAICRN initiated a clinical laboratory quality improvement program in the region. This included: (i) baseline assessment of each hospital clinical laboratory against international standards, ( ii) inventory, maintenance, and calibration of instruments, (iii) enrollment in external quality assurance program, (iv) assessment of training needs, (v) review of normal reference values used in the laboratory, and (vi) accreditation status of the laboratories by local and international accreditation bodies. The baseline assessments evaluated the overall quality of the clinical laboratory and helped to develop short- and long-term recommendations for improvement. Based on the assessment and recommendations, subsequent follow-up visits and technical support were provided to each hospital to implement those recommendations.

Laboratory management staff in each hospital were trained and encouraged to implement a quality management system. This included development and implementation of quality and technical manuals, standard operating procedures (SOPs), and a document control system. To help to improve laboratory quality it was necessary to appoint a senior staff member as a Quality Officer to oversee all aspects of laboratory quality and the quality enhancement program. Furthermore, each laboratory was supported with necessary instruments, and staff were encouraged to establish and monitor an equipment maintenance and calibration program. All staff from each laboratory were trained on standards developed by the technical committee of the International Organisation for Standardisation (ISO15189:2003 and ISO15190:2003, http://www.iso.org/) [10].

During the clinical trials it was noticed that occasionally reported biochemistry results were implausible. In response, a continuous improvement system was implemented to ensure that laboratory test results are reviewed by a qualified person to identify performance issues. In addition, advice was given on proper use of internal controls, how to monitor and investigate the possible cause(s) of controls not meeting acceptance criteria, and corrective and preventive action measures when performance issues are identified. Laboratories were encouraged to complement the capacity of each other by establishing a specimen testing referral linkage among them.

The overall activities of the SEAICRN created opportunities for laboratory staff to exchange experiences with senior laboratory personnel. In addition, a laboratory networking system and a platform for sharing experiences among laboratories was established through the SEAICRN annual meetings and regular teleconferences. Also, as part of the development of new clinical protocols there has been a strong interaction between laboratory experts, clinicians, and trial support staff. Having such systems in place have proved crucial to the ability of these clinical laboratories to respond quickly to the spread of oseltamivir-resistant influenza A/H1N1 and pH1N1. With the infrastructure in place it proved possible to respond immediately to the pH1N1 pandemic. It was also possible to implement a clinical research protocol in Viet Nam and start to make the data available in less than a month from the appearance of the first cases.

The ultimate goal of the program was to improve the quality of laboratory services, achieve compliance with GCLP standards, gain ISO 15189 accreditation, and facilitate better clinical care and collaborative research. Setting such goals makes the process more real and motivates laboratory staff to participate. Several laboratories in Thailand had already started working toward accreditation independently of the SEAICRN, and these efforts helped facilitate the SEAICRN program at those sites. Two laboratories in Thailand received “The Association of Medical Technologists of Thailand” accreditation, and one laboratory in Thailand and two in Vietnam have gained ISO15189 accreditation. In addition, another laboratory in Thailand and one in Indonesia are nearly ready for accreditation inspection.

## Discussion

Although GCLP is the standard in developed countries, it can pose a disproportionate burden and significant challenge in resource-constrained settings (see Box 1). However, it was clear that laboratory staff were motivated to improve the quality of their testing and services when working toward a concrete endpoint, such as ISO15189 accreditation. A GCLP standard for resource-constrained settings that is simpler and less demanding on resources is urgently needed [5],[11].

**Box 1. Key Challenges**

1. Improving laboratory capacity requires investment in both time and money. It is essential that long term goals are set, as opposed to short term fixes, and that the finances are made available to enable and sustain continuous quality improvements.
2. Laboratories will be at different stages of implementing an international-level quality system. A capacity building program should be tailored to each laboratory taking cultural, technical, and financial differences into account. Working towards accreditation is a concrete and achievable goal and helps to motivate staff.
3. International accreditation (e.g. ISO15189:2003) may not always be desirable and sustainable in resource-constrained settings as the ongoing costs may be too high. It is worthwhile to explore alternate accreditation models in such settings, which can be used as a stepwise approach to full international accreditation.
4. Improving laboratory quality systems requires financial commitment and support from the hospital director and a time commitment from laboratory leadership and staff. All need to be convinced that improvement is essential for delivering good quality health care. Research activities should not be solely responsible for funding the running costs of quality systems as this would not be sustainable with the systems potentially ending when the research funding stops.
5. Not all countries have accreditation authorities that can accredit laboratories as qualified people are not available. The capacity for national or regional accreditation should be improved in the near future.

It is important that laboratory enhancement programs focus not only on the particular disease of research interest, so that the investments can also benefit other diseases and routine patient care [12]. Setting up the SEAICRN has served as a catalyst for laboratories to implement molecular diagnostic techniques as part of routine care, not only for influenza but for a range of other pathogens beyond direct research needs. The SEAICRN has provided tools, training (including short courses, Masters, and PhD programs), and protocols to facilitate these processes. Currently, six scientists from Asia are enlisted in a PhD program, nine in a Masters program, three in a Bachelor's program, and 295 in a short-term fellowship. A total of 2,146 individuals have participated in operational training activities organized or made possible by the SEAICRN.

The sharing of data has been a contentious issue over the last few years. The SEAICRN has a policy that all the protocols, case record forms, SOPs, and manuals of operations for all studies and for all processes are made available via the internet at the start of all studies (http://www.seaicrn.org/). During the pH1N1 pandemic, a number of non-Asian countries used these protocols, adapted them to their own local circumstances, including translation, and implemented them. SEAICRN is also committed to sharing all data with all institutions and national and international authorities, in line with the IHR 2005. However, it is also crucial for scientists and clinicians within countries and within regions to know each other, to have built sustainable relationships and networks in a manner that encourages and facilitates the exchange of experience and information within the regional clinical and scientific community.

Clearly, the success and sustainability of a research network depends on funding. The current support for the SEAICRN ends in September 2010, but investigators across the region are committed to continuing the network, and funding has been secured to ensure its sustainability beyond that date. There is a clear commitment to extend the network beyond the current centers in the four countries in Asia. Other centers have expressed an interest in joining or supporting such an expansion. The long-term aim is to build a sustainable clinical research network with its center of gravity firmly based in Asia.

Having a wider perspective has proven invaluable. Furthermore, the SEAICRN was able to respond immediately in assessing oseltamivir resistance in seasonal influenza A/H1N1 in 2008, providing vital information to the scientific and medical community. We believe this program has improved the clinical and research laboratory capacity and quality in the region. Besides enhancing research in Asia, the investments have also helped improve health care for all patients served at the participating hospitals. Our program addresses the issues raised by the World Health Assembly in 2005 and in the revised International Health Regulations, as it supports the region to be more prepared to deal with potential pandemic influenza viruses and other endemic and emerging infectious diseases, as we have recently experienced, and promotes international collaboration and sharing of data.

## Abbreviations

GCLP: good clinical laboratory practice
MDL: molecular diagnostic laboratory
pH1N1: influenza A (H1N1)
RT-PCR: reverse transcriptase polymerase chain reaction
SEAICRN: South East Asia Infectious Disease Clinical Research Network
SOP: standard operating procedure
